# Plasma Rich in Growth Factors (PRGF) Increases the Number of Retinal Müller Glia in Culture but Not the Survival of Retinal Neurons

**DOI:** 10.3389/fphar.2021.606275

**Published:** 2021-03-09

**Authors:** Noelia Ruzafa, Xandra Pereiro, Alex Fonollosa, Javier Araiz, Arantxa Acera, Elena Vecino

**Affiliations:** ^1^ Experimental Ophthalmo-Biology Group, Department of Cell Biology and Histology, University of Basque Country UPV/EHU, Leioa, Spain; ^2^ Begiker-Ophthalmology Research Group, Cruces Hospital, BioCruces Health Research Institute, Bilbao, Spain; ^3^ Department of Ophthalmology, University of Basque Country UPV/EHU, Leioa, Spain; ^4^ Biodonostia Health Research Institute, Donostia Hospital, San Sebastian, Spain

**Keywords:** retina, retinal disease, PRP, PRGF, inflammation, cytokines, neuron, glia

## Abstract

Plasma rich in growth factors (PRGF) is a subtype of platelet-rich plasma (PRP) that stimulates tissue regeneration and may promote neuronal survival. It has been employed in ophthalmology to achieve tissue repair in some retinal pathologies, although how PRGF acts in the retina is still poorly understood. As a part of the central nervous system, the retina has limited capacity for repair capacity following damage, and retinal insult can provoke the death of retinal ganglion cells (RGCs), potentially producing irreversible blindness. RGCs are in close contact with glial cells, such as Müller cells, that help maintain homeostasis in the retina. In this study, the aim was to determine whether PRGF can protect RGCs and whether it increases the number of Müller cells. Therefore, PRGF were tested on primary cell cultures of porcine RGCs and Müller cells, as well as on co-cultures of these two cell types. Moreover, the inflammatory component of PRGF was analyzed and the cytokines in the different PRGFs were quantified. In addition, we set out to determine if blocking the inflammatory components of PRGF alters its effect on the cells in culture. The presence of PRGF compromises RGC survival in pure cultures and in co-culture with Müller cells, but this effect was reversed by heat-inactivation of the PRGF. The detrimental effect of PRGF on RGCs could be in part due to the presence of cytokines and specifically, to the presence of pro-inflammatory cytokines that compromise their survival. However, other factors are likely to be present in the PRGF that have a deleterious effect on the RGCs since the exposure to antibodies against these cytokines were insufficient to protect RGCs. Moreover, PRGF promotes Müller cell survival. In conclusion, PRGF hinders the survival of RGCs in the presence or absence of Müller cells, yet it promotes Müller cell survival that could be the reason of retina healing observed in the *in vivo* treatments, with some cytokines possibly implicated. Although PRGF could stimulate tissue regeneration, further studies should be performed to evaluate the effect of PRGF on neurons and the implication of its potential inflammatory role in such processes.

## Introduction

As a part of the central nervous system (CNS), the retina has a limited capacity for repair after disease or lesion. Different diseases affect retinal ganglion cells (RGCs), the neurons responsible for communication between the eye and the brain. RGCs die by apoptosis in glaucoma, a neurodegenerative disease that provokes irreversible blindness (Glovinsky et al., 1991; Garcia-Valenzuela et al., 1995; Quigley et al., 1995; Quigley, 2011). However, they can also die following axon degeneration (Knoferle et al., 2010), ischemia (Selles-Navarro et al., 1996; Joo et al., 1999) or in diabetes (Lieth et al., 2000; Zhang et al., 2000). Nevertheless, it has been seen that RGCs can recover their regenerative capacities in appropriate environments (Garcia et al., 2002; Berry et al., 2008; Ruzafa and Vecino, 2015; Vecino et al., 2015).

Plasma rich in growth factors (PRGF), a subtype of P-PRP (pure platelet-rich plasma), is a supernatant enriched in plasma and platelet-derived morphogens, proteins and growth factors. PRGF represents a complex pool of active mediators that may stimulate and accelerate tissue regeneration, which is generally safe to use and inexpensive to obtain. Indeed, autologous PRGF has been approved for clinical use by the European Community and the U.S. Food and Drug Administration (Anitua et al., 2014b), and it is generally employed in ophthalmology as eye drops to treat the ocular surface (Lopez-Plandolit et al., 2010; Lopez-Plandolit et al., 2011). Furthermore, different PRPs are being used in clinic: autologous platelet injections are being used to treat recurrent retinal detachment (Nadal et al., 2012) and pilot studies are being carried out to use PRGF in retinal surgery to treat persistent macular holes (Arias et al., 2019). Although visual acuity may be improved in patients, these treatments could be associated with complications such as focal macular epithelial pigmentary hypertrophy, retinal folds emanating from the macula, development of epiretinal membrane or cataract progression (Cheung et al., 2005; Nugent and Lee, 2015). Thus, although PRGF has been successfully used, the events that could be triggered by PRGF in the retina are not completely understood.

An intranasal delivery system has been used with human PRGF to reverse neurodegeneration and rescue memory in a transgenic mouse model of Alzheimer’s disease (AD). Moreover, PRGF can exert a neurotrophic effect in response to amyloid beta and promote neuronal survival (Anitua et al., 2015a). Since PRGF could offer neuroprotection and it is approved for use in clinical practice, we set out to study if PRGF can protect RGCs and enhance the survival of these cells.

RGCs are in close contact with Müller glia, the main glial cells in the mammalian retina. These cells that serve to maintain retinal homeostasis, and they are involved in retinal metabolism, in the phagocytosis of neuronal debris, in the release of certain transmitters and trophic factors, as well as in K^+^ uptake (reviewed in (Vecino et al., 2016)). Müller cells extend across the thickness of the retina, providing structural stability and maintaining close contact with the majority of retinal neurons (Bringmann and Reichenbach, 2001). In addition to their involvement in maintaining homeostasis, these cells also provide trophic factors to neurons that potentially promote their survival and repair (Bringmann et al., 2006; Reichenbach and Bringmann, 2013), and they have been seen to enhance RGC survival (Garcia et al., 2002; Ruzafa et al., 2018). Therefore, in the CNS, PRGF may interact with these glial cells as it contains growth factors known to accelerate cell proliferation, stimulate differentiation and promote cell survival (O'kusky et al., 2000; Jin et al., 2002). Among the growth factors present in PRGF, those implicated in proliferation include platelet-derived growth factor (PDGF), transforming growth factor beta (TGF-β), vascular endothelial growth factor (VEGF), fibroblast growth factor (FGF), epidermal growth factor (EGF), insulin-like growth factor I (IGF-I) and nerve growth factor (NGF: (Orive et al., 2009).

In light of the neuroprotective and pro-survival properties of PRGF, and knowing that PRPs are being used to treat retinal disorders (Nadal et al., 2012; Arias et al., 2019); we aimed to determine whether PRGF stimulates Müller cell survival and if it provides neuroprotection to RGCs. The effect of the PRGF on these two cell types were studied separately and together in a well-established adult culture system (Garcia et al., 2002; Garcia and Vecino, 2003; Ruzafa and Vecino, 2015; Vecino et al., 2015; Pereiro et al., 2020). In addition, since PRGF contains molecules with other activities and some that are implicated in immune responses (Anitua et al., 2015b), we set out to define the cytokines that could be implicated in the effects in PRGF. Finally, in order to relate the presence of cytokines in PRGF with its effect on retinal cells, we set out to suppress the possible pro-inflammatory response of PRGF through heat inactivation, or by adding the anti-inflammatory drug dexamethasone or antibodies against the three major pro-inflammatory cytokines (IL-1β, IL-6, and TNFα), studying how the inhibition of the inflammatory response affects RGCs.

## Materials and Methods

### Study Design

**FIGURE 1:**
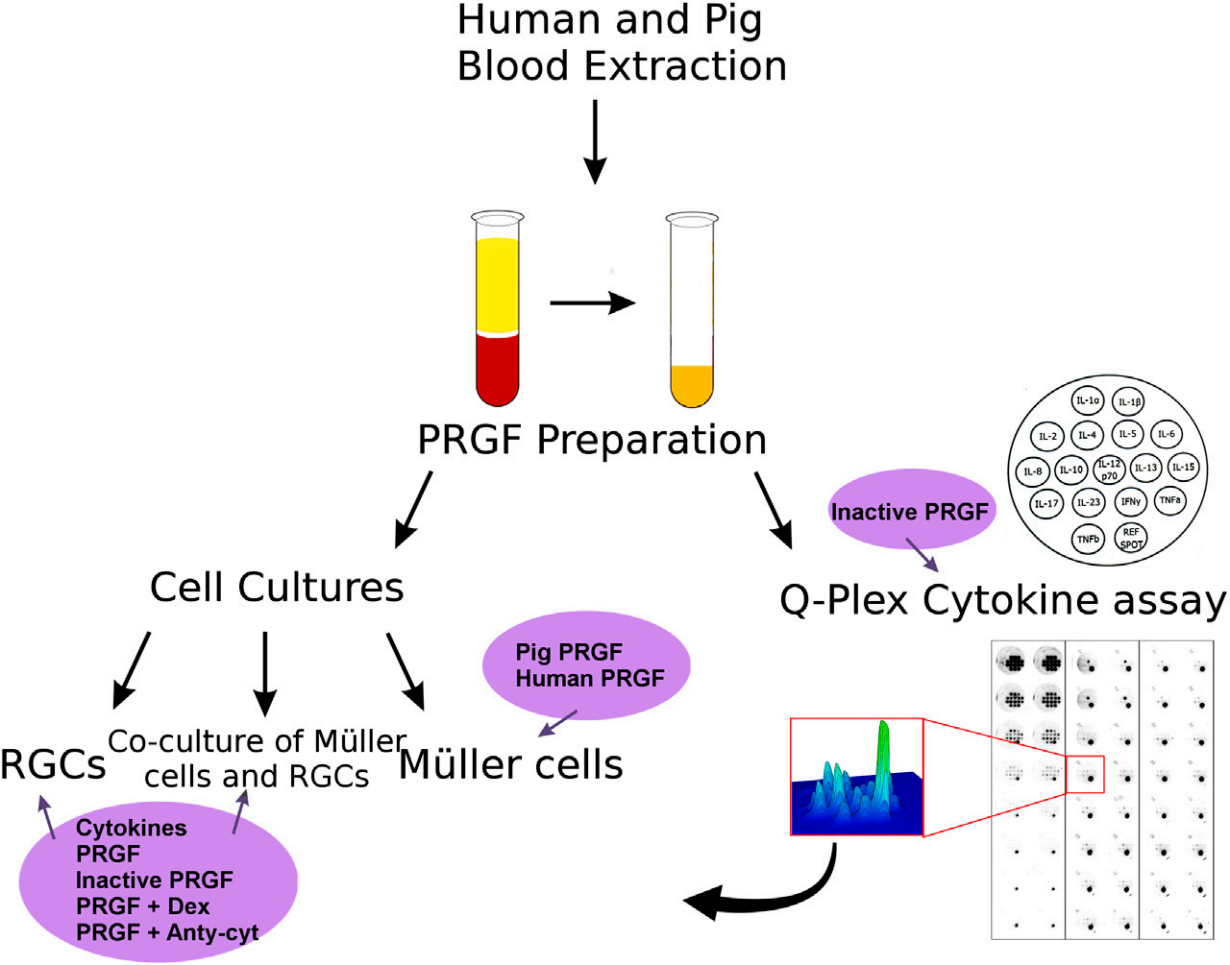
Scheme of the analysis of the properties of the PRGF and its effects on retinal cell cultures performed in this study. First, human and porcine blood was extracted and PRGF was obtained by plasma extraction. The effect of the PRGF was analyzed on three different types of cell cultures: pure RGCs or Müller cell cultures, and co-cultures of both these cell types. In addition, the presence of cytokines in the human and porcine PRGF was quantified through a Q-plex assay. The heat-inactivated form of PRGFs were also analyzed. After confirming the presence of cytokines in the PRGF, we checked the effect of the presence of pro-inflammatory cytokines (IL-1β, IL-6, and TNFα) on the RGCs cultures and co-cultures. We heat-inactivated the PRGF, and we added the inflammatory drug dexamethasone (Dex) or antibodies against cytokines (anti-cyt) to the PRGF in order to analyze its effect on the cell cultures. The survival effect of porcine and human PRGF on pure Müller cells cultures were also assessed.

On the present study, the effect of the PRGF was analyzed on different retinal cell cultures and the cytokines in the PRGF were quantified (Figure 1).

### Porcine Samples

Adult porcine eyes (*n =* 20) and blood (*n =* 5) were obtained at a local slaughterhouse and the eyes were transported to the laboratory on ice in CO_2_-independent medium (Life Technologies, Carlsbad, CA, United States) with 0.1% gentamicin. The retinas were obtained from the eyes 1–2 h after enucleation. All animal experimentation adhered to the ARVO Statement for the Use of Animals in Ophthalmic and Vision Research.

### Human and Pig PRGF

This study was carried out by qualified personnel in strict accordance with the tenets of the Helsinki Declaration on Biomedical Research Involving Human Subjects. Before blood collection, signed informed consent was obtained from all subjects once the nature of the study and the possible consequences of the study had been explained to them. Human blood samples were obtained through antecubital vein puncture and PRGF was obtained as described previously (Anitua et al., 2015a), with some minor modifications. Briefly, human (*n =* 3) and porcine (*n =* 5) blood was collected in 5 ml tubes containing 3.8% (wt/vol) sodium citrate. Samples were centrifuged at 460 g for 8 min at room temperature and the plasma fraction containing platelets was separated, avoiding the buffy coat and erythrocytes. The plasma fraction (1 ml) was reconstituted for 1 h at 34°C with 50 µl calcium chloride (Braun Medical, Melsungen, Germany) in glass tubes, and the supernatant released was collected after centrifugation at 460 g for 15 min. Finally, part of the total volume of the PRGF obtained was heat-inactivated at 56°C for 60 min, following a previously published protocol (Anitua et al., 2014a), and both the samples (PRGF and inactive PRGF) were filtered through a filter with a 0.2 μm pore size of (Fisher Scientific, Madrid, Spain), aliquoted and stored at −80°C.

### Cell Culture

Retinal cell cultures were prepared according to the method reported previously (Garcia et al., 2002; Ruzafa et al., 2018), with the following minor modifications. Three types of cultures were used: 1) RGCs cultured in B27-supplemented Neurobasal-A medium (Life Technologies, Carlsbad, CA, United States); 2) a co-culture of RGCs and Müller cells in B27-supplemented Neurobasal-A medium with 10% fetal bovine serum (FBS: Life Technologies, Carlsbad, CA, United States); and 3) Müller cell cultures in DMEM (Life Technologies, Carlsbad, CA, United States) supplemented with 10% FBS. 1% L-glutamine (2 mM) and 0.1% gentamycin (50 mg/ml) were added to all media.

The retinas were dissected out and 8 mm diameter pieces were obtained with a dissecting trephine (Biomedical Research Instruments, MD, United States), avoiding the most peripheral retina and visible blood vessels. The tissue was disrupted enzymatically with papain at 37°C (Worthington Papain Dissociation kit, Worthington Biochemical Lakewood, NJ, United States) for 90 min in the presence of 10% DNAse I (Worthington Papain Dissociation kit, Worthington Biochemical Lakewood, NJ, United States) to obtain RGCs, or for 30 min to obtain Müller cells and for co-cultures. Papain activity was stopped by adding medium and the tissue was disaggregated by gentle trituration using pipette tips of decreasing diameter. Dissociated retinal cells were collected by a 5 min centrifugation at 300 g and resuspended in medium. The RGC culture was prepared following the protocol of the Worthington Papain Dissociation kit (Worthington Biochemical Lakewood, NJ, United States), plating the resuspended cells in twenty four well plates on 13 mm poly-L-lysine (100 μg/ml: Sigma, P4832) and laminin (10 μg/ml: Sigma, L2020) coated glass coverslips. The cells were maintained in a humidified incubator at 37°C in an atmosphere of 5% CO_2_. For the Müller cell cultures and the co-cultures, the entire medium was changed on day 1 and to maintain all the cells, half of the medium was replaced every 3 days. The cells were fixed for 10 min with methanol at -20°C after 7 days, before the Müller cells cultures reached confluence. At least three replicates of each culture were studied and the procedures were carried out in triplicate.

PRGF (10%) was added at the beginning of the cell culture and maintained throughout, and for the Müller cell cultures PRGF was added in the absence of FBS. To analyze the effect of PRGF in Müller cells, non-autologous porcine and human PRGF were used. The RGCs and the co-cultures were subjected to different experimental conditions (Table 1), using only human PRGF due to the difficulties in obtaining porcine PRGF.

**TABLE 1:** Different experimental conditions to which the RGC cultures and the co-cultures of RGCs and Müller cells were exposed.

Control (0% PRGF)
Cytokines (IL-1β, IL-6 and TNFα)
10% Human PRGF
10% Inactive Human PRGF
10% Human PRGF + Dex
10% Human PRGF + anti-cytokines

Dexamethasone (Dex, 1 μM: Sigma Aldrich, St. Louis, MO, United States) was added to the cultures, or a mix of the three major pro-inflammatory cytokines at a concentration of 10 ng/ml each (as recommended by (Madeira et al., 2015): IL-1β, IL-6 and TNFα (Sigma-Aldrich, St. Louis, MO, United States). To inactivate these pro-inflammatory cytokines, antibodies against these cytokines were added to the cultures (at 1 μg/ml, the concentration recommended by (Madeira et al., 2015): goat anti-IL-1β and mouse anti-IL-6 (R&D System, Minneapolis, MN, United States), and rabbit anti-TNFα (Peprotech, London, United Kingdom) antibodies). The cytokines, in the presence or absence of their antibodies, were also added from the beginning of the cultures.

### Immunochemistry

The cells were fixed in methanol, washed with PBS (phosphate-buffered saline, pH 7.4) and immunostained as described previously (Vecino et al., 2002). After blocking non-specific antigens with blocking buffer (3% BSA and 0.1% Triton X-100 in PBS) the cultures were exposed to the following antibodies: a rabbit polyclonal anti-βIII-tubulin antiserum (diluted 1:2,000: Promega, Madison, WI, United States), an RGC marker; and mouse monoclonal anti-vimentin antibody (diluted 1:10,000: Dako, Glostrup, Denmark) as specific marker for Müller cells. After again washing in PBS, antibody binding was detected with the following secondary antibodies (diluted 1:1,000): anti-rabbit Alexa Fluor 555 and anti-mouse Alexa Fluor 488 (Life Technologies, Carlsbad, CA, United States). The cells were also stained with the DAPI nuclear marker at a dilution of 1:10,000 (Life Technologies, Carlsbad, CA, United States).

### Cell Quantification

The surfaces of the whole 13 mm diameter coverslips were analyzed and at least three replicates of each culture condition were analyzed, performing these experiments in triplicate. The images were taken with an epifluorescence microscope (Zeiss, Jena, Germany) coupled to a digital camera (Zeiss Axiocam MRM, Zeiss, Jena, Germany) and using the Zeiss Zen software (Zeiss, Jena, Germany). A mosaic of the entire coverslip was analyzed using 359, 488, and 555 nm filters with a 10X objective, and once the mosaic was defined, the coverslip surface area was calculated (132.73 mm^2^). The Zeiss Zen software (Zeiss, Jena, Germany) was used to count the Müller cell nuclei and for the mosaic definition, and the RGC density was also analysed. The cell density was calculated as the mean number of cells/mm^2^, and the data were normalized to the control to simplify its representation.

### Multiplex Cytokine Assays

To detect and quantify the cytokines present in the different PRGF samples, a multiplex enzyme-linked immunosorbent assay (ELISA) was used (Q-Plex™, Human Cytokine Screen, 110996HU, Quansys Bioscience, Logan, UT, United States), measuring: IL-1α, IL-1β, IL-2, IL-4, IL-6, IL-8, IL-10, IL-12p70, IL-13, IL-15, IL-17, IL-23, TNFα, and TNFβ. The assay was performed according to the manufacturer´s instruction, analysing 100 μl of the different human and porcine PRGF samples (pig, inactive pig, human, inactive human) in each well of a 98-well plate Q-Plex™. The standards were measured in duplicate and the cytokine concentrations were calculated using a standard curve. All samples were assessed in four replicates and arithmetic averages were calculated.

### Statistical Analyses

Statistical analyses were carried out using the SPSS Statistics software v. 21.0 (IBM) and, the mean and the standard error for each condition were calculated. The data from the different experimental conditions were compared using an analysis of variance (ANOVA), followed by the Games-Howell test as the variances were not homogeneous according to a Levene test. For the multiplex cytokine assays, a non-parametric Kruskal-Wallis H test was used. The minimum value accepted for significant differences in all the tests was defined as *p* < 0.05.

## Results

The inflammatory cytokines in the pig and human PRGF were assessed, quantifying the following cytokines in these PRGF samples: IL-1α, IL-1ß, IL-2, IL-4, IL-6, IL-8, IL-10, IL-12, IL13, IL-15, IL-17, IL-23, TNFα and TNFß (Table 2).

**TABLE 2:** Concentration (pg/ml) of the cytokines in active and inactive porcine and human PRGF

Cytokine	Pig PRGF	Inactive pig PRGF	Human PRGF	Inactive human PRGF
**IL-1α**	8.15 ± 1.67	10.31 ± 0.87	6.34 ± 2.18	6.55 ± 2.39
**IL-1ß**	14.65 ± 4.93	20.91 ± 7.06	17.03 ± 4.35	17.01 ± 2.54
**IL-2**	4.58 ± 1.15	6.93 ± 0.13	4.54 ± 0.54	6.17 ± 1.66
**IL-4**	0.29 ± 0.28	0.87 ± 0.70	0.39 ± 0.32	0.17 ± 0.17
**IL-6**	3.61 ± 0.49	5.21 ± 0.18	4.24 ± 1.08	3.99 ± 1.14
**IL-8** *	1.32 ± 0.42	1.24 ± 0.05	29.59 ± 5.73	29.21 ± 3.38
**IL-10**	4.65 ± 1.47	6.85 ± 2.65	7.21 ± 1.52	4.73 ± 1.61
**IL-12**	4.96 ± 1.11	5.86 ± 2.11	5.00 ± 3.87	6.71 ± 3.90
**IL-13**	0.37 ± 0.21	0.29 ± 0.22	0.75 ± 0.75	0.35 ± 0.20
**IL-15**	2.29 ± 0.49	4.54 ± 1.23	7.09 ± 2.79	6.77 ± 1.92
**IL-17**	4.59 ± 0.21	2.93 ± 1.02	1.68 ± 0.99	2.88 ± 0.97
**IL-23**	185.47 ± 43.63	242.71 ± 86.04	283.14 ± 214.27	222.54 ± 158.70
**TNFα**	4.65 ± 4.25	3.76 ± 3.76	6.61 ± 2.99	1.84 ± 1.84
**TNFß**	18.40 ± 4.64	14.07 ± 9.76	18.15 ± 14.21	14.88 ± 13.96

* (significant differences between pig and human PRGF).

When the cytokine concentrations in pig and human samples were compared, only the IL-8 concentration was significantly higher in the human PRGF than in the pig PRGF (*p* < 0.001). Moreover, heat inactivation did not alter the cytokine concentrations in either the pig or human PRGFs (their activity was not assessed). As some individual variability was detected in the quantification of the cytokines, we analysed the differences in the cytokine concentrations between individuals. We detected significant differences between individuals for the IL-8, IL-12, IL-13, IL-15, IL-17, IL-23 and TNFα (*p* < 0.05) in the human PRGF, and for the IL-1ß, IL-10, IL-15, IL-17 and IL-23 (*p* < 0.05) in the pig PRGF. The remaining cytokines were relatively homogeneous between individuals, with no significant differences detected.

**FIGURE 2:**
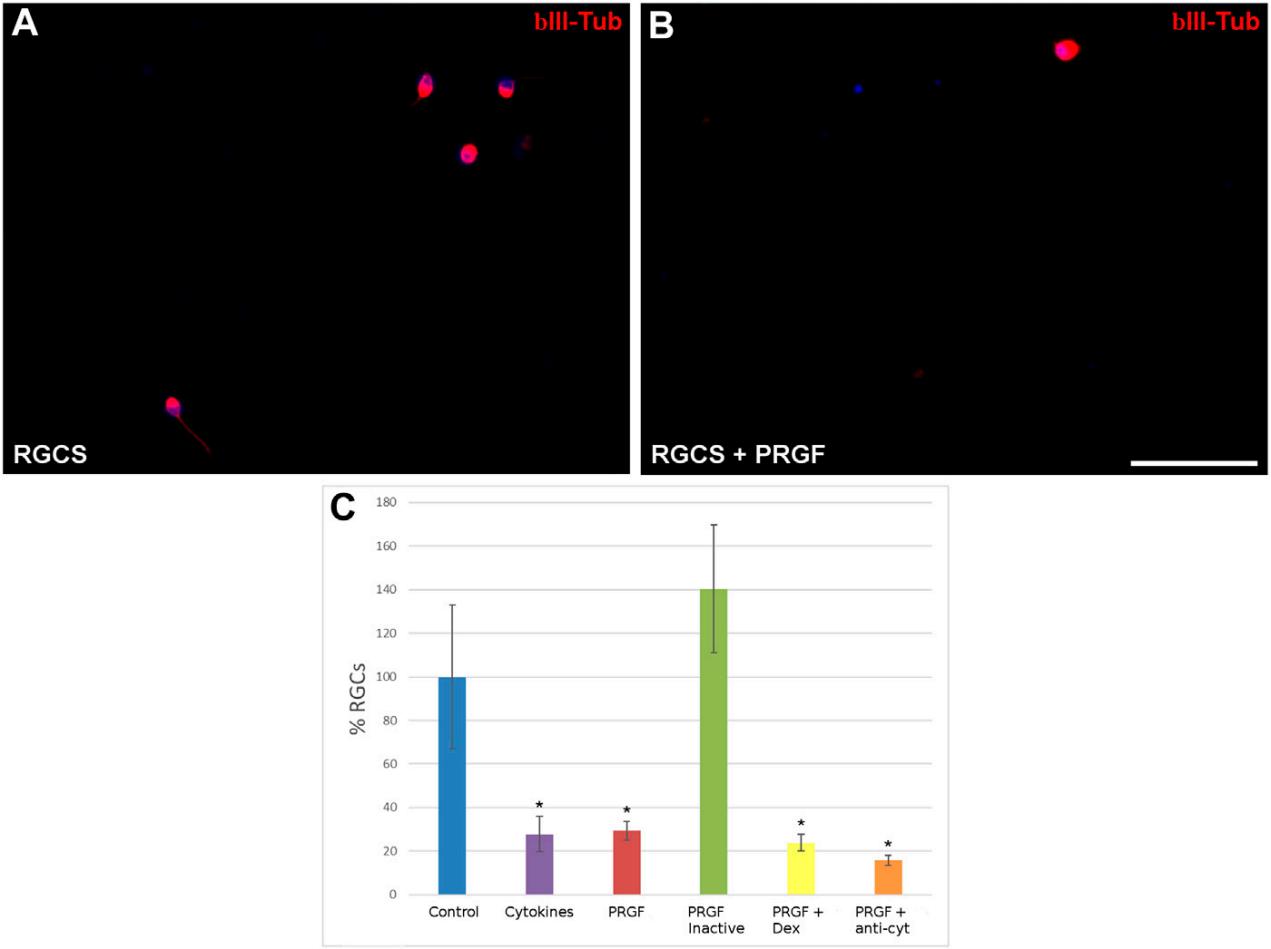
The effect of PRGF on RGC cultures. Images of control adult pig RGC cultures **(A)** or RGCs maintained in the presence of PRGF **(B)**. The RGCs were labeled with antibodies against βIII-Tubulin (red) staining the nuclei with DAPI (blue). The percentage of RGCs **(C)** relative to the controls are represented in the histograms. RGCs were cultured in the presence of cytokines (IL-1β, IL-6, and TNFα), 10% human PRGF (PRGF), PRGF inactivated by heat, PRGF with dexamethasone (Dex) and PRGF with antibodies against cytokines (anti-cyt). Significant differences relative to the controls are shown:*p < 0.05. Scale bar = 100 μm.

In light of the cytokines detected in the PRGF, their neuroprotective effects were analyzed by initially quantifying the density of RGCs in the presence or absence of 10% human PRGF (Figure 2). The density of RGCs was 0.55 ± 0.11 cells/mm^2^ in control cultures, considered as 100%, yet a significant decrease in RGC density was evident in the presence of PRGF (29.29 ± 4.31%, *p* < 0.05), similar to that detected when the cultures were exposed to the pro-inflammatory cytokines IL-1β, IL-6 and TNFα (27.80 ± 8.03%, *p* < 0.05). Heat inactivation of the PRGF did not affect to RGC survival (140.36 ± 29.29%), favoring the survival of these cells to a similar extent as that seen in the control cultures. However, the presence of the inflammatory drug dexamethasone (23.76 ± 3.76%) or of antibodies against the pro-inflammatory cytokines (15.69 ± 2.35%) did not reverse the deleterious effect of PRGF on RGCs, provoking similar survival of RGCs to the PRGF alone (*p* < 0.05, Figure 2C). Thus, these cytokines could be at least partially responsible for the detrimental effect on the PRGF as RGC survival diminished in their presence alone. However, others factors in the PRGF must also have a deleterious effect on the RGCs, as in the presence of the antibodies against these cytokines the survival of RGCs was still compromised.

**FIGURE 3:**
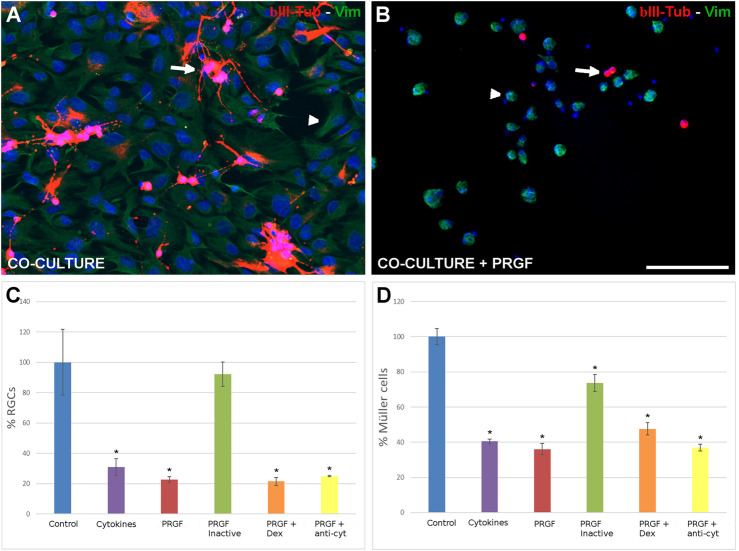
The effect of PRGF on RGCs and Müller cells in co-culture. Control co-cultures of adult pig RGCs and Müller cells **(A)** and those maintained in the presence of PRGF **(B)**. The RGCs (arrows) were labeled with antibodies against βIII-Tubulin (red) and Müller cells (arrowheads) with antibodies against vimentin (green) and the nuclei were labeled with DAPI (blue). The number of RGCs **(C)** and Müller cells **(D)** in the co-cultures relative to the total number of cells in the control condition (100%) are represented in the histograms. RGCs and Müller cells were co-cultured with cytokines (cyt: IL-1β, IL-6 and TNFα), 10% human PRGF (PRGF), heat inactivated PRGF, PRGF with dexamethasone (Dex) and PRGF with antibodies against the cytokines (anti-cyt). Significant differences relative to the control conditions are shown: *p < 0.05. Scale bar = 100 μm.

Since Müller cells possess neuroprotective properties and given that PRGF enhances their number, we assessed the effect of PRGF on the RGCs co-cultured in the presence of Müller cells in co-cultures (Figures 3A,B). Interestingly, PRGF had a similar effect on RGCs in co-cultures as in the pure RGC cultures. In the presence of Müller cells, the RGC density still decreased in the presence of PRGF (22.67 ± 1.91%) or cytokines (30.89 ± 5.49%) relative to the control conditions (12.97 ± 1.33 RGCs/mm^2^, 100%: *p* < 0.05). The heat inactivation of PRGF abrogated its deleterious effects on RGCs (92.22 ± 8.11%), the survival of which was no different to the controls. However, the presence of dexamethasone (21.54 ± 2.65%) or the presence of antibodies against the cytokines (24.99 ± 0.28%) did not revert the deleterious effects of PRGF on RGCs, diminishing their survival relative to the controls (*p* < 0.05, Figure 3C).

In terms of Müller cells, the increase in their number in the presence of 10% human PRGF was dampened when they were co-cultured with RGCs (36.09 ± 3.31%) and relative to the control conditions (209.29 ± 28.43 Müller cells/mm^2^ as 100%, *p* < 0.05: Figure 3B). This decrease in the number of Müller cells was similar to the witnessed in the presence of the cytokines alone (40.43 ± 1.24%). Moreover, heat inactivation (73.56 ± 4.8%), the presence of dexamethasone (47.61 ± 3.46%) or of the antibodies against the cytokines (36.96 ± 1.88%) did not revert the effect of PRGF and the density of Müller cells remained lower than in the control cultures (*p* < 0.05). This difference in the behavior of Müller cells in the presence of PRGF is probably due to the inclusion of FBS in the co-culture medium, which in these co-cultures had to be maintained for the cells to grow correctly.

**FIGURE 4:**
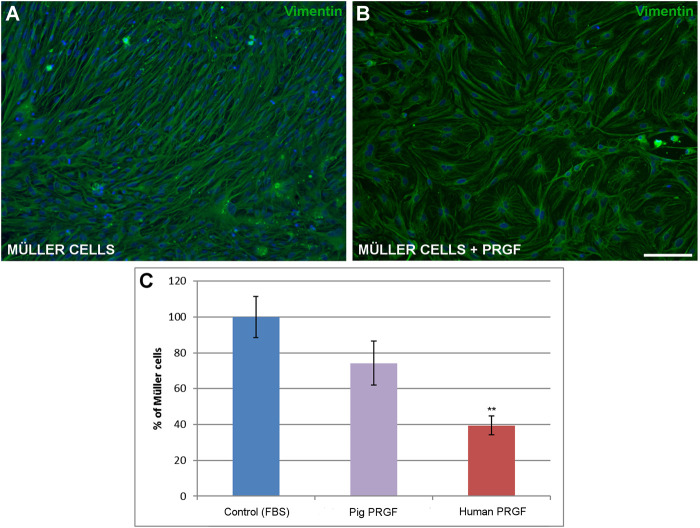
The effect of PRGF on Müller cell density. Images of adult pig Müller cells cultured in the absence **(A)** or in the presence of PRGF **(B)**. The Müller cells were labeled with antibodies against vimentin (green) and the nuclei with DAPI (blue). The number of Müller cells represents the percentage **(C)** relative to the total number of Müller cells in the control conditions (100%). Müller cells were cultured with 10% fetal bovine serum (FBS) in control, 10% pig PRGF or 10% human PRGF. Significant differences of control and human PRGF are shown: **p < 0.01. Scale bar = 100 μm.

Finally, to assess the influence of PRGF on the survival of Müller cells, the density of Müller cells in the pure cultures was evaluated in the presence or absence of pig and human PRGF. We compared the density of Müller cell in positive control where 10% FBS was added to the medium (569.69 ± 59.85 cells/mm^2^, considered as 100%), as opposed to those in which the medium was supplemented with 10% pig or human PRGF. DMEM medium without either the FBS or PRGF supplement served as the negative control, was not increase Müller cell survival. The density of Müller cells maintained in the presence of PRGF was less than when these cells were maintained in the presence of FBS, although in both cases the cultures reached confluence (Figures 4A,B). When the 10% FBS was replaced by 10% porcine PRGF there was no significant difference in the density of Müller cells (74.21 ± 12.19%) relative to the controls, whereas the Müller cell density did decrease significantly in the presence of 10% human PRGF (39.44 ± 5.34%, *p* < 0.01) relative to the control conditions (100%, Figure 4C). Nevertheless, the substitution of FBS with PRGF induced an increase of Müller cells, and Müller cells failed to proliferate or survive in the absence of either PRGF or FBS.

## Discussion

PRGF provides tissues with a pool of growth factors, suggesting that it may stimulate and accelerate tissue regeneration (Anitua et al., 2015b). For this reason, PRGF is considered a potential therapeutic agent suitable to treat neurodegenerative diseases Anitua et al., 2015a). However, in this study on retinal cells, we found that PRGF reduced the number of retinal neurons, such as RGCs, while promoting the increase of Müller glial cells.

The proliferative effects of PRGF were first demonstrated in dentistry and oral implantology, orthopedics, in the treatment of skin disorders and sports medicine (Sánchez et al., 2003). Indeed, several studies have described the potential of PRGF eye drops to produce tissue regeneration in the ophthalmological field, where it has advantages over other PRPs (Lopez-Plandolit et al., 2010; Lopez-Plandolit et al., 2011). Our results demonstrate that PRGF may induce an increase in Müller cells density. Porcine PRGF is capable of substituting serum when Müller cells are cultured alone. This is likely to be due to the growth factors present in the PRGF, such as IGF-1, PDGF or FGF, powerful stimulators of cell replication and proliferation (Vahabi et al., 2015).

The ability to induce cells division of PRGF is not only evident in the pig PRGF but also, to a lesser extent in the human PRGF that partially activated the division of porcine Müller cells, showing that this effect of PRGF could be interspecific, although the pig PRGF increased higher the number of Müller cells than the human PRGF. This behavior is similar in different types of cells, because different PRPs, including PRGF, induce a proliferative response (Vahabi et al., 2015), as seen here in the Müller cells in the absence of serum. Moreover, different platelet preparations produce similar proliferative effects on immortalized human Müller cells (MIO-M1) (Burmeister et al., 2009). In addition, intravitreal injection of PRPs induces pre-retinal proliferation of fibroblast-like cells (Pinon et al., 1992) and these fibroblast-like cells could be Müller cells. Currently, autologous platelet injections are being used in the clinic as a treatment for recurrent retinal detachment (Nadal et al., 2012), and pilot studies are being carried out to use PRGF in retinal surgery to treat persistent macular holes (Arias et al., 2019), showing benefits in patients but possibly also associated with some complication including focal macular epithelial pigmentary hypertrophy, retinal folds emanating from the macula, development of epiretinal membrane, among others (Minihan et al., 1997; Cheung et al., 2005; Nugent and Lee, 2015). The success in the clinical uses of PRPs, such as PRGF, could be due to their proliferative effects on cells like Müller glia.

It has also been suggested that PRGF induces neuroprotection by activating the anti-apoptotic PI3K/Akt signaling pathway, and/or through reducing caspase-3, promoting proliferation and survival in primary neuronal cultures. In addition, PRGF has been proposed to reduce the number of degenerating neurons in a mouse model of AD, where it dampens astrocyte reactivity, prevents the loss of synaptic proteins and stimulates global improvements in anxiety, learning and memory behaviors (Anitua et al., 2014b). However, our data show PRGF compromises the survival of RGCs, even when they are co-cultured with Müller cells. It is important to note that in these earlier studies, PRGF obtained from human blood was used in mouse models (Anitua et al., 2015a) and thus, interspecific interactions did not appear to affect neuroprotection. However, we have founded that the porcine and human PRGF did not exert the same effect on the cultures as was mentioned previously. Moreover, the concentrations of PRGF used here were 10%, the same as those used in these aforementioned studies of neurodegenerative diseases (Anitua et al., 2014b; Anitua et al., 2015a), although these were *in vivo* studies and the behavior of the retinal cells in cultures may differ in response to the same concentration of PRGF. Although the plasma concentrate is distinct, some results are consistent with our findings, where other PRPs may produce neurotoxic effects, such as thrombin-activated platelet-rich plasma, possibly due to the release of glutamate in neuronal cultures (Bell et al., 2014; Zhou et al., 2017). Moreover, PRGF contains many different factors that are also present in PRP, including morphogens and growth factors (Anitua et al., 2015b), and that exert negative effects on axon growth in rat brain/spinal cord co-cultures, such as TGF-β1 (Takeuchi et al., 2012). These mechanisms could partially explain the results obtained here on RGCs.

In co-cultures, the negative effect of PRGF on RGCs survival was observed as well as when they were maintained in pure cultures. Müller cells provide structural support, maintain retinal homeostasis, promote the survival of neurons, and secrete neurotrophins and growth factors that offer protection to neurons (Garcia et al., 2003; Vecino et al., 2016). Thus, since PRGF increases Müller cell number, we expected that the survival of RGCs would be enhanced in co-culture thanks to their possible neuroprotective action. However, the presence of Müller cells was not sufficient to rescue RGCs from the detrimental effects of the PRGF. In addition, the Müller cells density was weaker in the co-cultures in presence of PRGF, possibly due to the extra effort of these cells to maintain the RGCs alive or to other mechanisms not assessed here. In addition, we observed that the morphology of the cells dramatically change when they are in co-culture in the presence of PRGF. The decrease in the survival of both Müller cells and RGCs suggest that both types of cells are stressed or even dying. In the first steps of the apoptosis, there is a shrinkage of the cells (Saraste and Pulkki, 2000) that correspond to the rounded morphology of Müller cells in this condition.

In order to better understand the effect of PRGF on RGCs and Müller glia, we analyzed the cytokines that it contains. Some cytokines have already been defined in PRGF, like IL-6 (Masuki et al., 2016), IL-8, IL-4 and TNFα (Anitua et al., 2015b), yet here we confirmed the presence of the 16 selected cytokines analyzed in five porcine and three human samples: IL-1α, IL-1β, IL-2, IL-4, IL-5, IL-6, IL-8, IL-10, IL-12p70, IL-13, IL-15, IL-17, IL-23, IFNγ, TNFα and TNFβ. Moreover, there were no significant differences in the cytokine concentrations between porcine and human PRGF, except for the higher abundance of the pro-inflammatory cytokine IL-8 in human PRGF.

Despite the wide range of cytokines identified in the PRGF, the presence of the pro-inflammatory cytokines IL-1α, IL-1β and IL-2 is particularly noteworthy due to their possible contribution to the neurotoxic and proliferative effects of PRGF. The neurotoxic effect of IL-1 is evident through an increase in neuronal apoptosis in the hippocampus and dentate gyrus (Rincon-Lopez et al., 2016), and in the retina (Kido et al., 2001). Moreover, the neurotoxicity of IL-1β could be specifically mediated by glial cells (Thornton et al., 2006), and/or to enhanced endoplasmic reticulum stress as a result of the disturbance of intracellular Ca^2+^ homeostasis provoked via NMDARs (Dong et al., 2017). In addition, IL-2 may also be neurotoxic and produce nervous tissue damage (Hanisch et al., 1997). However, these interleukins have also been implicated in proliferation and IL-1 can stimulate diverse cell types (Rangnekar et al., 1991; Jerde and Bushman, 2009). Indeed, IL-1α can stimulate proliferation of neural progenitor cells (Mcpherson et al., 2011) and IL-1β significantly augments smooth muscle cell proliferation (Zhai et al., 2004). Furthermore, IL-2 can increase the proliferation of T lymphocytes (Pesoa et al., 2000) or other cell types like enterocytes (O'loughlin et al., 2001). However, we do not rule out the possibility that there are other molecules in addition to these that could be implicated in these effects. Moreover, microglial cells may also be implicated in the effect of PRGF in the retina as we have described a migration of these cells as a sing of inflammation in presence of PRGF. (Ruzafa et al., 2021).

The presence of the three major pro-inflammatory cytokines (IL-1β, IL-6 and TNFα (Taki et al., 2007; Zhang and An, 2007) in the cultures could compromise RGC survival due to the neurotoxic effect that they can exert (Downen et al., 1999; Ye et al., 2013). However, the suppression of their effects by adding antibodies against these cytokines to the PRGF previous to be added to the cultures was insufficient to prevent RGC death. Thus, the mechanism implicated in neuronal death triggered by PRGF appears to be multifactorial. On the other hand, RGC death can be reversed by the heat-inactivation of PRGF, which maintains the biological activity of PRGF but completely reduces the complement activity and significantly deceases the presence of IgE (Anitua et al., 2014a). Although the inactivated PRGF apparently contains the same cytokines as the active PRGF, and in similar amounts, heat-inactivated PRGF is a safer format to administrate this therapy, especially for subjects affected by immune disorders, due to the cytokines activity, that was not assessed here, may have been compromised. This response could be due to the reduction in neuronal viability by complement (Peterson et al., 2017), the inhibition of which could be neuroprotective (Yang et al., 2013) and sufficient to protect RGCs from death. In addition, it should be noted that although inflammatory pathways are implicated in neuronal death, the presence of the anti-inflammatory drug dexamethasone does not enhance the RGCs survival.

Although PRGF has been successfully used in different medical and surgical specialties, and there is extensive data indicating that PRP induces tissue regeneration, many of these studies may not have been sufficiently rigorous or well-controlled, and their data is often limited, whereas other studies have yielded contrasting results (Kuffler, 2015). The impact exerted by PRGF is as yet not fully understood, such as its neurotoxic effect, and as such its application in ophthalmology must be studied in greater depth. Indeed, the deleterious effect of PRGF on RGCs demonstrated in our study should be taken into account when using it to treat retinal disorders like macular holes. Though a structural recovery might be achieved, for instance macular hole closure, probably due to the promotion of increases Müller cell number, the functional recovery might be limited due to its toxic effects on RGCs. The differences in the concentrations of the multitude of factors in PRGF and other PRPs may explain the variability in the results obtained between different studies (Vahabi et al., 2015). Indeed, we found significant differences in cytokine concentrations between individuals and thus, further animal and clinical studies should be performed to clarify the full range of properties of PRGF.

In conclusion, PRGF does not offer neuroprotection to RGCs but rather, it markedly compromises the survival of RGCs, even when these cells are co-cultured with Müller cells. However, this effect could be reverted by heat-inactivating the PRGF. Conversely, PRGF increases Müller cell number, and it may be a good candidate to stimulate and accelerate tissue regeneration due to these properties. These responses could be produced by the presence of several cytokines in PRGF, such as the IL-1α, IL-1β and IL-2 that are present in both human and pig PRGF. Although the factors responsible for promoting glial survival in the PRGF could be the neurotrophins identified previously, other molecules could also participate in this effect. Therefore, further studies will be necessary to clarify the effect of PRGF in the nervous system, as well as the other properties of PRGF, including the implication of the cytokines it contains.

## Data Availability

The raw data supporting the conclusions of this article will be made available by the authors, without undue reservation.
